# The phylogenetic signal in tooth wear: What does it mean?

**DOI:** 10.1002/ece3.4541

**Published:** 2018-10-09

**Authors:** Larisa DeSantis, Mikael Fortelius, Frederick E. Grine, Christine Janis, Thomas M. Kaiser, Gildas Merceron, Mark A. Purnell, Ellen Schulz‐Kornas, Juha Saarinen, Mark Teaford, Peter S. Ungar, Indrė Žliobaitė

**Affiliations:** ^1^ Vanderbilt University Nashville Tennessee; ^2^ Department of Geosciences and Geography University of Helsinki Helsinki Finland; ^3^ Departments of Anthropology and Anatomical Sciences Stony Brook University Stony Brook New York; ^4^ University of Bristol Bristol UK; ^5^ University of Hamburg Hamburg Germany; ^6^ PALEVPRIM UMR 7262 CNRS INEE & Université de Poitiers Poitiers France; ^7^ Centre for Palaeobiology Research School of Geography, Geology and the Environment, University of Leicester Leicester UK; ^8^ Max Planck Weizmann Center for Integrative Archaeology and Anthropology, Max Planck Institute for Evolutionary Anthropology Leipzig Germany; ^9^ Touro University Vallejo California; ^10^ University of Arkansas Fayetteville Arkansas; ^11^ Department of Computer Science and Finnish Museum of Natural History University of Helsinki Helsinki Finland

**Keywords:** mesowear, microwear, phylogenetic methods, tooth wear

## Abstract

A new study by Fraser et al (2018) urges the use of phylogenetic comparative methods, whenever possible, in analyses of mammalian tooth wear. We are concerned about this for two reasons. First, this recommendation may mislead the research community into thinking that phylogenetic signal is an artifact of some sort rather than a fundamental outcome of the evolutionary process. Secondly, this recommendation may set a precedent for editors and reviewers to enforce phylogenetic adjustment where it may unnecessarily weaken or even directionally alter the results, shifting the emphasis of analysis from common patterns manifested by large clades to rare cases.

Dear Editor,

A new study by Fraser, Haupt, and Barr (2018) urges the use of phylogenetic comparative methods, whenever possible, in analyses of mammalian tooth wear. We are concerned about this for two reasons. First, this recommendation may mislead the research community into thinking that phylogenetic signal is an artifact of some sort rather than a fundamental outcome of the evolutionary process. Secondly, this recommendation may set a precedent for editors and reviewers to enforce phylogenetic adjustment where it may unnecessarily weaken or even directionally alter the results, shifting the emphasis of analysis from common patterns manifested by large clades to rare cases.

Fraser et al. (2018) test for phylogenetic signals in diet and tooth wear of mammals and find that the dependence between diet and phylogeny is extremely strong. Not surprisingly, they also find strong dependence between tooth wear proxies and phylogeny. What they fail to emphasize is that this makes complete sense: If a phylogenetic signal was present in diet but absent in dietary proxies, it would imply that proxies have nothing to do with diet. Reassuringly, such is not the case.

When diet is related to phylogeny, any proxy will inevitably incorporate some of that phylogenetic signal. The more accurate the proxy, the better it will capture the true diet and the stronger will be the phylogenetic signal because both are linked at several levels of adaptation. This does not indicate that the proxy itself is phylogenetically biased, and it just may reflect different facets of this link.

Consider a hypothetical example of a simple world consisting only of two extant orders: Carnivora and Artiodactyla. In this system, diet would be nearly maximally correlated with phylogeny, since those eating meat would be mostly in Carnivora, while plant eaters would be mostly in Artiodactyla. Suppose we use the presence of carnassial teeth as a proxy for inferring diet. In the simple world of Carnivora and Artiodactyla, all of the meat eaters and extremely few of the plant eaters (such as the giant panda) would have carnassials. The dietary proxy, thus, would be maximally correlated with phylogeny, because diet is maximally correlated with phylogeny. This by no means implies that the presence of carnassials would not be a reliable indicator of diet. Carnassials do have a common phylogenetic origin, but they have been retained in so many species because of their function, not in spite of it. Indeed, many other carnivorous species (mainly extinct ones) have convergently derived carnassial dental morphology with the type of shearing wear seen in the carnassials of carnivorans, both among placentals (e.g. oxyaenids and hyaenodontids) and marsupials (thylacines, thylacoleonids, and many sparassodontids).

Fraser et al. (2018) take phylogenetic niche conservatism as a starting point for their study, implying that species are constrained by ancestral traits and therefore have a “reduced dietary ability over evolutionary time.” That is plausible, as per our hypothetical example above. However, what is misleading is the follow‐up statement that “dietary inferences from tooth wear are biased by phylogenetic relatedness,” inferred from the fact that “tooth wear dietary proxies show strong phylogenetic signal”.

Methods of determining diet from tooth wear, as well as any other analytical methods of determining diet such as stable isotopes or fecal analyses, do not assume or require diet to be independent of phylogenetic affinity. What they assume is that within the domain of validity of a method, each diet leaves a detectable trace (i.e. it wears teeth in a distinct way or leaves a distinct composition of isotopes), no matter which taxon the tooth comes from. This gives these proxies the flexibility to not only monitor similarities in diet when they might be expected (e.g. between certain distantly related taxa), but to document differences in diet when they might not necessarily be expected based on tooth morphology alone. Differences between closely related taxa have been captured, for instance, in bovids (Scott, 2012; Ungar, Merceron, & Scott, 2007), cervids (Berlioz, Kostopoulos, Blondel, & Merceron, 2017), ungulates (Schulz, Calandra, & Kaiser, 2010), feliforms (DeSantis & Haupt, 2014; DeSantis, Tseng, et al., 2017), canids (DeSantis et al., 2015), primates (Scott et al., 2005; Ungar, Grine, & Teaford, 2008), and macropodids (DeSantis, Field, Wroe, & Dodson, 2017; Prideaux et al., 2009). Indeed, many bioarchaeological studies have demonstrated distinctive and predictable diet‐related differences in both gross dental wear and microwear within a single species, *Homo sapiens* (Rose & Ungar, 1998).

The *Equus* and *Bos* example, pointed out by Fraser et al (2018) as a case of possible problem with phylogenetic non‐independence, in fact shows the opposite: Distantly related taxa, with convergently similar diet, have very similar results from all dental wear proxies (Kingston, 2011). Further, experimental studies have demonstrated different microwear attribute values in the same species when fed foods with different textural properties and/or grit loads (e.g. Schulz et al., 2013; Merceron et al., 2016). In many cases, the diets of extinct taxa are also vastly different from those of extant taxa, despite being members of the same family (e.g. Kaiser, 2011, Mihlbachler, Rivals, Solounias, & Semprebon, 2011; DeSantis, Schubert, Scott, & Ungar, 2012).

Function‐driven methods, such as mesowear or microwear, therefore give us the chance to recognize both similarities and differences in paleobiology that might not be detectable via traditional morphological or taxic techniques. The same sort of thing can be said for stable isotope techniques where, for instance, experimental manipulations of diet result in changes to the isotopic composition of sampled tissues (e.g. DeNiro & Epstein, 1978; Passey et al., 2005) and where, for example, carbon isotope values for bovids from the early hominin sites in South Africa indicate that 25% of specimens would be misclassified (in terms of diet) if based on taxonomic affiliation alone (Sponheimer, Reed, & Lee‐Thorp, 2001).

The fact that diet is not independent of phylogeny is one of the fundamental outcomes of the evolutionary process, since a descendent species is never created de novo but builds upon inheritance from its immediate ancestor. “Species of the same genus have usually, though by no means invariably, some similarity in habits and constitution, and always in structure” (Darwin, 1872 p 60), this is a phylogenetic signal. Phylogenetic niche conservatism, however, results when closely related species are more similar ecologically than would be expected based on their phylogenetic relationships (Losos, 2008). Therefore, the phylogenetic signal itself, as tested by Fraser et al (2018), would not indicate evolutionary constraints; however, its residual as compared to random divergence could indicate it (see Revell, 2010 for more details).

Multiple studies (reviewed by Losos, 2008) have found that phylogenetic niche conservatism is by no means omnipresent, while phylogenetic signal is the norm. And even if lineages remain conserved within their diets and habitats, it does not follow that functional relationships between (dental) traits and diets become decoupled. If teeth of the same kind remain useful for the same diets, like carnassials for eating meat, inference of diets via dental proxies would not be biased.

An analogy can be made to an X‐ray machine used at airports to detect the presence of sharp objects. Suppose a family arrives that have jointly prepared for a picnic, and therefore, all have knives in their pockets. If the machine correctly detects all the knives, it would yield the equivalent of a phylogenetic signal in its outputs. This does not mean that the method by which the machine detects knives is biased, or needs to be adjusted. And if we adjust the machine in order to decrease phylogenetic signal, in this situation, the result will be undoubtedly worse; the machine will either make false alarms or miss real detections (Type I and Type II errors).

Tooth wear is a function of behaviors occurring in life. It is fundamentally different from genetically mediated dietary proxies, such as occlusal morphology, so its analysis should not be constrained by the use of phylogenetic comparative methods.

We do not mean to imply that phylogenetic methods are wrong as such. They have their meaningful uses but their relevance depends on the research question rather than the source of the data. Yet a statement received by some of us from a recent reviewer echoes a worrying trend to enforce phylogenetic adjustments everywhere where data of phylogenetic origin are used: “Although I agree with the authors’ rationale for not wanting to use PGLS^1^, in this day and age it is simply not acceptable to NOT do the analyses using PGLS.” We argued that this was unnecessary, and the editors were in agreement.

Analysis of phylogenetically related data is not trivial, and there is no solution that fits all purposes (see Westoby, Leishman, and Lord (1995), Losos (2008), Revell (2010) for comprehensive discussions). We strongly disagree with the recommendation of Fraser et al (2018) to use phylogenetic comparative methods in studies of mammalian tooth wear whenever possible. Phylogenetic regression is not by itself a better or more advanced analysis method than ordinary regression. The choice of the methods and interpretations of results depends on the authors of analyses and the research questions they tackle.

## AUTHORS’ CONTRIBUTIONS

LD initiated the collaborative response. IZ wrote up the first draft. All the authors contributed to the final version. The authorship order was assigned alphabetically.

## DATA ACCESSIBILITY

This commentary has no associated data.

## ENDNOTES

1 One of the methods for eliminating phylogenetic dependencies, phylogenetic generalized least squares.
